# *Aggregatibacter aphrophilus* chronic lacrimal canaliculitis: a case report

**DOI:** 10.1186/s12886-016-0312-3

**Published:** 2016-08-02

**Authors:** Marie Boulze-Pankert, Cécile Roux, Vanessa D. Nkamga, Frédérique Gouriet, Marie-Christine Rojat-Habib, Michel Drancourt, Louis Hoffart

**Affiliations:** 1 Service d’Ophtalmologie, Hôpital de la Timone, Aix-Marseille-Université, Marseille, France; 2 Fédération de Microbiologie Clinique et Unité des Rickettsies, CNRS UMR 6020, Aix-Marseille Université, IHU Méditerranée Infection, Marseille, France; 3 Service d’anatomopathologie, Hôpital de la Timone, Marseille, France; 4 Unité de Recherche sur les Maladies Infectieuses et Tropicales Emergentes, Faculté de Médecine, 27, Boulevard Jean Moulin, 13385 Marseille Cedex 5, France

**Keywords:** Canaliculitis, *Aggregatibacter aphrophilus*, *Haemophilus aphrophilus*, diagnosis, Canaculitis, *Aggregatibacter aphrophillus*, Canaliculotomy, Case report

## Abstract

**Background:**

Chronic canaliculitis is often misdiagnosed as conjunctivitis, delaying proper documentation and management. *Aggregatibacter aphrophillus* has not been implicated in chronic canaliculitis.

**Case presentation:**

We report a case of unilateral chronic epiphora associated with chronic lacrimal canaliculitis resistant to prolonged topical antibiotic treatment in a 65-year-old woman without notable medical history. Canaculotomy, curettage with removal of concretions and tubing with silicone stent for six weeks resolved this chronic infection. Culturing lacrimal secretions and concretions yielded *Aggregatibacter aphrophilus* in pure culture. Histological analyses showed elongated seed clusters surrounded by neutrophils. Fluorescence in Situ Hybridization confirmed the presence of bacteria in two distinctive concretions.

**Conclusion:**

This first documented case of *A. aphrophilus* chronic lacrimal canaliculitis illustrates that optimal surgical management of chronic lacrimal canaliculitis allows for both accurate microbiological diagnosis and treatment.

## Background

Chronic canaliculitis is often misdiagnosed as conjunctivitis, delaying proper management. Its diagnosis should include appropriate microbiological investigations based on the analysis of surgical clinical specimens, as treatment should include both surgery of the canaliculus and pathogen-targeted antibiotic treatment. Based on this approach, we here report on a case of *Aggregatibacter aphrophilus* chronic canaliculitis, firmly diagnosed using advanced microbiological methods.

## Case presentation

**Fig. 1:**
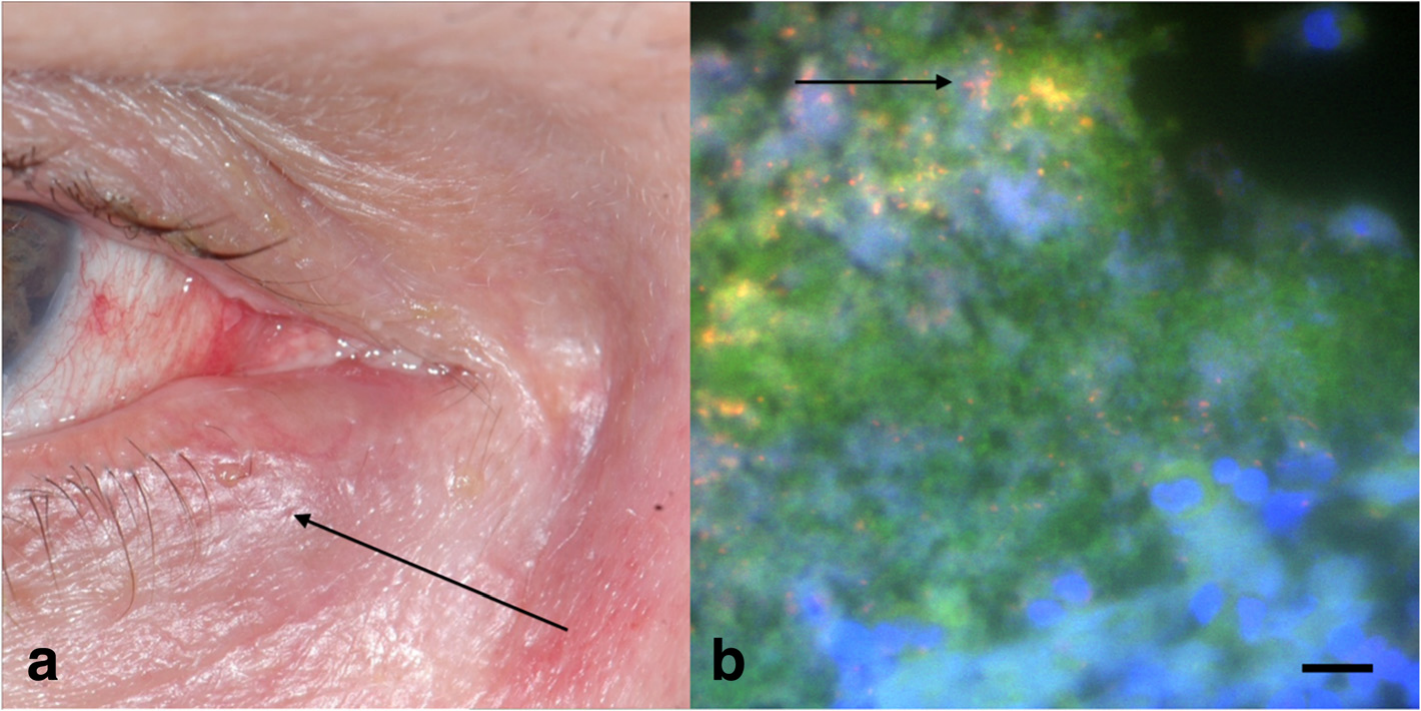
Chronic *Aggregatibacter aphrophilus* lacrimal canaliculitis. **a** Photography of right eye showing swollen lower canaliculis (arrow), inflamed conjunctiva and mucopurulent secretions (**b**) FISH detection of *A. aphrophilus* in canaliculitis concretions. Fluorescent microscopy revealed bacteria labeled by EUB338 probe (red fluorescence) (arrow) when combining non-specific DNA label by DAPI (blue fluorescence) and negative control probe (green fluorescence). Scale bar, 50 microns

A 65-year-old woman was referred to perform a dacryocystorhinostomy for chronic epiphora with mucopurulent secretions resistant to several topical antibiotic treatments. The patient had no history of lacrimal plug, palpebral surgery or trauma. This patient had been initially diagnosed with chronic conjunctivitis and dacryocystis. However, her clinical presentation included a lower eyelid erythema and a red, pouting punctum expressing a mucopurulent discharge after bidigital massage (Fig. 1a). Slit lamp examination showed pericanalicular inflammation without lacrimal sac involvement. Probing and irrigation showed permeability of the lacrimal drainage system. Chronic canaliculitis was finally diagnosed and the patient underwent canaliculotomy under general anaesthesia involving a linear incision into the conjunctival side of the canaliculus, curetting of concretions and tubing with a silicone stent (Mini Monoka silicone stent, FCI Ophthalmics, Paris, France) for six weeks; followed by topical dexamethasone 1 mg/mL combined with tobramycin 0.3 % QID for 15 days. Culture of the secretions and concretions on 5 %-sheep blood Colombia agar incubated under a strict anaerobic atmosphere for seven days remained sterile but culture on a chocolate agar (PolyViteX, bioMérieux, Marcy l’Etoile, France) incubated in a 5 % CO_2_-enriched atmosphere yielded *Aggregatibacter aphrophilius* identified by matrix-assisted laser desorption ionization time-of-flight mass-spectrometry (MALDI-TOF-MS) with an identification score of 1.737. Using the disk diffusion method, the isolate tested susceptible to amoxicillin (minimum inhibitory concentration (MIC), 0.5 mg/L), ceftriaxone (MIC <2 g/L), gentamicin (MIC, 0.25 mg/L) and rifampicin (MIC <2 g/L). The microbial community structure of the canaliculitis was studied by Fluorescence in situ hybridization (FISH) incorporating probe EUB338 5′-GCTGCCTCCCGTAGGAGT-3′ labeled with Alexa fluor-546, specific for Eubacteria 16S rRNA gene and probe ARC915 5′-GTGCTCCCCCGCCAATTCCT-3’ labeled with Alexa fluor-488, specific for Archaea 16S rRNA gene. FISH revealed cocci detected by EUB338 probe (red fluorescence), while the ARC915 probe (green fluorescence) remained negative (Fig. 1b). Histological analysis after hematoxylin and eosin staining showed clusters of elongated microorganisms surrounded by neutrophils. After ablation of the silicone stent at six weeks, the four-month follow-up showed no sign of infection and the patient was judged cured.

## Conclusions

Chronic lacrimal canaliculitis is rarely detected in clinical practice, accounting for 2 % of lacrimal duct diseases. This inflammation is caused by infection or punctual plug insertion. Generally, canaliculitis is a primitive unilateral condition caused by *Streptococcus* spp., *Staphylococcus* spp. or *Actinomyces* spp. [1]. In the patient here reported, *A. aphrophilus*, formerly *Haemophilus aphrophilus*, a fastidious Gram-negative inhabitant of the oropharyngeal microbiota, was detected by FISH in two distinct concretions, cultured and firmly identified by mass spectrometry. Additional next-generation sequencing is a more research-oriented method, which can also be used in selected cases to disclose microorganisms. Only four cases of *A. aphrophilus* ocular infection have been previously reported [2–4] including two cases of endophthalmitis, one case of trabeculectomy bleb infection and one cited and as yet undescribed case of canaliculitis [3]. Other infections mainly include brain abscess [5] and endocarditis [6].

Topical antibiotics are ineffective for curing chronic canaliculitis due to chronic colonized concretions [7], as illustrated by the case here reported in which antibiotics failed, despite an exquisitely antibiotic-sensitive strain of *A. aphrophilus*. We therefore recommend surgical treatment, canaliculotomy with incision of the punctum and curetting of the concretions, as the standard treatment of chronic canaliculitis.

## Abbreviations

FISH, fluorescence in situ hybridization; MALDI-TOF-MS, matrix-assisted laser desorption ionization time-of-flight mass-spectrometry; MIC: minimum inhibitory concentration
